# Utilization of Dietary Supplements in People with the Atopic Triad in Korea: A Cross-Sectional Study Using KNHANES (2018–2021)

**DOI:** 10.3390/medicina61040718

**Published:** 2025-04-13

**Authors:** Hyunjoo Kim, Heesoo Bang, Eunkyung Euni Lee

**Affiliations:** Research Institute of Pharmaceutical Sciences, Natural Products Research Institute, College of Pharmacy, Seoul National University, Seoul 08826, Republic of Korea; mary_kim1@snu.ac.kr (H.K.); hban2031@snu.ac.kr (H.B.)

**Keywords:** allergic rhinitis, asthma, atopic dermatitis, atopic triad, population-based study, dietary supplements, KNHANES, national health survey, propolis

## Abstract

*Background and Objectives*: Studies investigating the types of dietary supplements associated with the atopic triad using large-scale data remain limited. We assessed the prevalence of the atopic triad, the types of dietary supplements used, and their associations in Korean adults using a 4-year national survey data. *Materials and Methods*: This cross-sectional study utilized data from the Korea National Health and Nutrition Examination Survey (2018–2021). Adults aged ≥ 19 years were included. Descriptive statistics were used to summarize participants’ overall characteristics and estimate the national prevalence of the atopic triad, defined as a diagnosis of asthma, allergic rhinitis, or atopic dermatitis. Logistic regression analyses were conducted using each atopic condition as a dependent variable, with the types of dietary supplements currently used as independent variables. Covariates included socioeconomic status, lifestyle factors, frailty, and diet. Dietary supplements were categorized as multivitamins/minerals, vitamin C, vitamin D, vitamin A/lutein, propolis, omega-3, probiotics, red ginseng, calcium, or iron. *Results*: A total of 18,182 adults were analyzed, representing an estimated 52.8 million adults (mean age: 47 years; 50% male). Of these, 21% reported a history of any atopic triad, and 61% were current supplement users. Logistic regression showed significantly higher odds of all three atopic conditions among users of propolis (asthma: OR 1.90, 95% CI 1.04–3.47; allergic rhinitis: OR 1.64, 95% CI 1.25–2.17; atopic dermatitis: OR 2.04, 95% CI: 1.18–3.52), and higher odds of allergic rhinitis among users of probiotics (OR 1.21, 95% CI 1.06–1.38) and vitamin D (OR 1.43, 95% CI 1.16–1.75). *Conclusions*: A positive association was found between propolis use and all three atopic conditions. Also, a positive association was found between probiotics or vitamin D use and allergic rhinitis. We did not find significant associations with the other supplements. Further research in establishing causal relationships between the dietary supplements and atopic conditions are needed.

## 1. Introduction

Asthma, allergic rhinitis, and atopic dermatitis are allergic disorders caused by abnormal immune responses characterized by type 2 inflammatory reactions and the release of various cytokines (e.g., IL-4, IL-5, IL-9, IL-13) [1]. They are commonly referred to as the “atopic triad” because they share a common genetic predisposition (e.g., filaggrin mutations and T helper type 2 [Th2] pathway genes) for allergic hypersensitivity, and individuals diagnosed with one condition are at increased risk of developing the others [2]. They are chronic disorders that persist into adulthood, often causing significant burdens such as increased healthcare costs and reduced quality of life [3,4]. Among these conditions, asthma has the highest mortality risk, causing approximately 250,000 deaths annually, mostly in low- and middle-income countries [5].

There is extensive literature on the potential protective role of dietary supplements in asthma and other allergic diseases which serve as a rationale for proposing these products in patients with allergic disease [6,7]. For example, observational studies have shown the potential protective role of vitamins C, E, selenium, magnesium, and iron, likely attributed to their antioxidant or anti-inflammatory properties [7,8,9]. However, findings from recent interventional studies remain controversial, especially in patients with asthma, with no evidence of benefit [7,10]. Other studies have also indicated excessive intake of some of these supplements such as iron, vitamin A, and propolis may exacerbate the allergic symptoms [11,12].

With the increasing popularity of dietary supplements, the benefits of their use are being raised as a subject of debate among researchers [11,12,13]. Nevertheless, the lack of population-based data on dietary supplements limits this evaluation. Therefore, we aimed to assess the prevalence of the atopic triad, the types of dietary supplements used in these patients, and further explore the potential associations between the atopic triad and specific types of dietary supplements using nationally representative survey data from South Korea. We hypothesized that certain dietary supplements may be associated with a higher prevalence of the atopic conditions, as some products have been associated with exacerbation of the symptoms. The findings of this study will provide real-world insights into the use of dietary supplements in these patients.

## 2. Materials and Methods

### 2.1. Study Population

This cross-sectional study utilized four consecutive years of data from the Korea National Health and Nutritional Examination Survey (KNHANES VII-VIII, 2018–2021), provided by the Korea Disease Control and Prevention Agency. Data were collected through interviews, health examinations, and surveys [14]. The survey employed a complex, multistage, clustered probability sampling design, which included sampling weights that allow for the generation of national-level estimates of the health status of non-institutionalized populations [14]. This study used secondary data and was approved by the Institutional Review Board (IRB) of Seoul National University (IRB No. E2502/003-009).

### 2.2. The Atopic Triad (Asthma, Allergic Rhinitis, Atopic Dermatitis)

Participants were considered to have a history of any atopic triad if they had ever been diagnosed with asthma, allergic rhinitis, or atopic dermatitis by a physician. Those who did not respond or selected ”No”, “Never diagnosed”, or “Don’t know” were categorized as non-atopic patients [14].

### 2.3. Dietary Supplements

Participants were categorized as current users of dietary supplements if they had taken them continuously for at least two weeks within the past year and were still taking them within the past two weeks, as per the KDCA KNHANES protocol [14]. If participants took dietary supplements in less than 2 weeks but planned to take them continuously, they were also considered current users [14]. Current users were asked to select a maximum of four types of dietary supplements [14].

### 2.4. Covariates

To minimize potential confounding bias, a comprehensive literature search was conducted to identify previously reported variables associated with the use of dietary supplements and allergic conditions [1,3,6]. Covariates were socioeconomic characteristics: sex, age groups in 10-year intervals, study years, marital status (0 = married and living together, 1 = never married, separated, widowed, or divorced), education (0 = did not graduate high school, 1 = graduated high school or above), living in a metropolitan statistical area (MSA), defined as top eight major cities in South Korea (0 = no, 1 = yes), equivalized household income (0 = Q2 or higher, 1 = Q1), and type of national insurance (0 = local/workplace, 1 = medical aid class 1 or 2, no health insurance, or unknown). We also incorporated other confounders previously reported to be associated with the use of dietary supplements or the atopic triad: a history of smoking (0 = never, 1 = current/past smoker) or drinking (0 = less than 4 times/month, 1 = at least 4 times/month last year), and frailty assessed using a 37-item Rockwood’s frailty index, with a score of 0.25 or higher defined as frailty (further details provided in Supplementary Table S1) [15]. The amount of nutrient intake from the diet, available from the 24-h recall data, was also adjusted for in the sensitivity analyses.

### 2.5. Statistical Analyses

All calculated estimates were weighted to represent the national population. Descriptive analyses were performed using chi-square or Student’s t-tests to summarize participants’ overall characteristics and the prevalence of atopic conditions. Unweighted frequencies, weighted means, percentages (%), and standard errors (SE) were presented. Logistic regression analyses were performed with each atopic condition as the dependent variable and specific types of dietary supplements as independent variables. The independent variables were not mutually exclusive as some participants used multiple dietary supplements. Covariates with a variance inflation factor higher than 5 were excluded. The distribution of continuous values was visually assessed to convert skewed data to a logarithmic scale for normalization. Odds ratios (OR) and 95% confidence intervals (CI) were presented.

Missing data were regarded as missing. All statistical analyses in our study followed the published guidelines for analysis by the KDCA [14]. All statistical analyses were performed using SAS version 9.4 (SAS Institute Inc., Cary, NC, USA), and the level of statistical significance was set at *p*-values < 0.05 (two-sided).

## 3. Results

**Figure 1:**
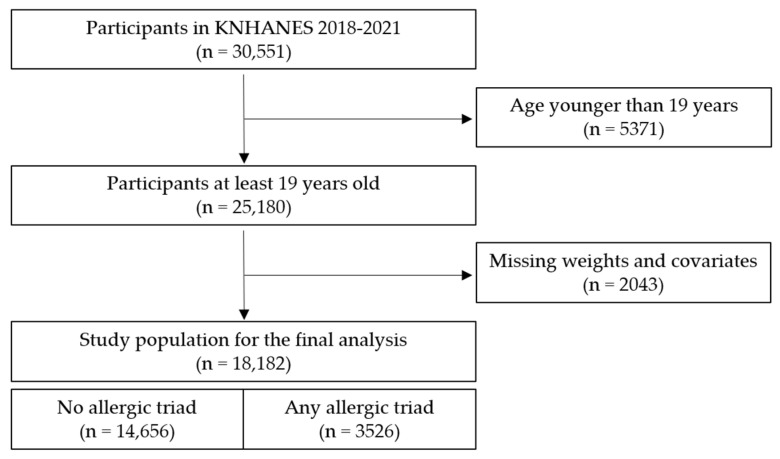
Identification of study population in KNHANES 2018–2021 (n, unweighted frequency).

A total of 30,551 men and women participated in the KNHANES 2018–2021. Of them, participants aged <19 years were excluded. Participants were also excluded if they did not participate in the dietary supplement surveys or if their covariate information was missing. Consequently, 18,182 participants were included in the final analysis, corresponding to 52,794,148 participants at the national level, representing 77% of Koreans at least 19 years old (Figure 1).

The mean (SE) age of the included adults was 47.31 (0.22) years, slightly less than half were men (49.96%), and approximately 61% were current supplement users (Table 1). Compared with the excluded adults of the same age group, those included were significantly younger, had a higher socioeconomic status, and were less frail. The proportion of supplement use, the number of concomitant supplements, and the prevalence of all three atopic conditions were higher in the included adults (Table S2). In addition, compared to 2018–2019, the prevalence of supplement use, allergic rhinitis, and atopic dermatitis, but not asthma, was significantly higher in 2020–2021 (Table S3).

**Figure 2:**
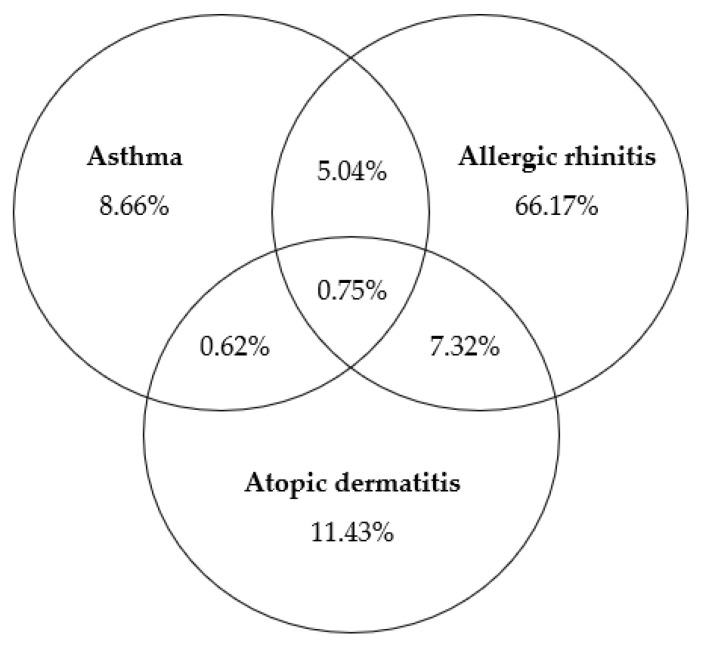
Venn diagram on weighted % of the atopic triad in adults 19+ years old in KNHANES 2018–2021.

In total, the prevalence of the atopic triad was 20.90% at the national level, with allergic rhinitis (16.57%) being the most common, while the other two conditions were each less than 5% (atopic dermatitis, 4.21%; asthma, 3.15%, Table S2). Most participants had been diagnosed with only one allergic condition (86.26%); among those with two or more, the combination of allergic rhinitis and atopic dermatitis was the most common (Figure 2).

Compared to adults with no atopic conditions, adults with atopic conditions were significantly younger, more likely to be women, had higher socioeconomic status, and were less frail (Table 1). These patterns were consistent when characteristics were compared by each atopic condition, except in asthma, where the opposite was observed (Tables S4–S6). The median age at diagnosis was also the highest in asthma (34.53 years), followed by allergic rhinitis (28.13 years) and atopic dermatitis (14.33 years) (Table S2).

From the multivariable logistic regression analysis, the prevalence of all three atopic conditions was 1.6 to 2.0 times higher in propolis users compared to non-users (asthma, OR: 1.90, 95% CI: 1.04–3.47; allergic rhinitis, OR: 1.64, 95% CI: 1.25–2.17; atopic dermatitis, OR: 2.04, 95% CI: 1.18–3.52; Table 2). Additionally, the prevalence of allergic rhinitis was 1.2 times higher in probiotic users (OR 1.21, 95% CI 1.06–1.38) and 1.4 times higher in vitamin D users (OR 1.43, 95% CI 1.16–1.75) (Table 2). Conversely, we did not find significant associations for the other supplements including multivitamins/minerals, omega-3, iron, vitamins A and C (Table 2).

The results from the multivariable logistic regression analysis remained consistent after additional adjustments for dietary nutrients from the previous day, with minimal increase in the adjusted ORs (asthma, OR: 1.85, 95% CI: 1.02–3.36, allergic rhinitis, OR: 1.63, 95% CI: 1.24–2.15; atopic dermatitis, OR: 2.01, 95% CI: 1.16–3.47; Table S7).

## 4. Discussion

In this cross-sectional study using national survey data, approximately 21% of Korean adults had a history of at least one of the allergic conditions in the atopic triad, with allergic rhinitis being the most common (17%). Furthermore, approximately 14% of adults had multiple atopic conditions, the most common combination being allergic rhinitis and atopic dermatitis. Among all dietary supplements, only propolis supplementation was associated with a higher prevalence of all three atopic conditions. We also found a higher prevalence of allergic rhinitis in users of probiotics and vitamin D supplements. These findings remained significant after rigorous adjustments for participants’ socioeconomic and lifestyle characteristics, frailty, and diet.

The prevalence estimates for the atopic triad reported in this study align with those previously reported: 17.1% for allergic rhinitis, 2.3% for asthma, and 3.9% for atopic dermatitis in Korean adults aged between 20 and 59 years from 2008 to 2017 [16]. The prevalence of allergic rhinitis is steadily increasing in Korea, with steeper slopes observed in older adults [16]. Adults with allergic rhinitis have at least three times higher risk of developing asthma [17]. These reports call for effective prevention and management strategies for chronic allergic diseases in the adult population [16]. Furthermore, we found that a history of the atopic triad was associated with higher socioeconomic status and a healthier lifestyle, as previously reported [17]. Several explanations exist for the higher prevalence in the resource abundant groups. One of them is the hygiene hypothesis stating exposure to allergens in early childhood helps develop a healthy immune system; thus, as people with higher socioeconomic status have lesser exposure to allergens, they are more likely to develop atopic conditions [17]. Or the higher prevalence could also indicate inflation in self-reported prevalence among wealthier individuals due to better access to healthcare and higher chances of receiving a diagnosis and being aware of the symptoms [17]. Adults with any atopic condition were also younger, which may be explained by immunosenescence in older adults [18]. Notably, contrary to the other two conditions, adults with asthma were older and had poorer socioeconomic status, which aligns with previous studies. For example, previously reported risk factors for asthma include residence in urban and high-poverty areas [19]. These findings highlight important differences in patient characteristics between asthma and the other two conditions.

In this study, we found that the prevalence of the atopic triad was 1.6 to 2.0 times higher in propolis users. Propolis is a bioactive phytochemical derived from hives and is rich in polyphenols and flavonoids [20]. Many preclinical studies have supported its anti-allergic properties, potentially by inhibiting mast cell and basophil activation [21]. Such findings have led to clinical trials of propolis supplementation in asthma [20,21]. For example, in a clinical study conducted in 46 adults with mild to moderate asthma, patients who took propolis with oral theophylline daily for two months experienced significant reductions in the frequency and severity of attacks, along with improvements in pulmonary function [22]. Similar benefits were also reported in another clinical trial involving 52 adults who received 75 mg of propolis tablets three times a day for one month [23]. For the other two conditions, however, there is currently a lack of clinical evidence supporting the benefit of propolis supplementation in adults [24,25]. Also, specific safety concerns have been reported regarding propolis supplementation, including increased risk of acute asthma, aspiration pneumonia, mediastinitis, and allergic dermatitis, necessitating evaluation of its safety in atopic patients who plan to use propolis [25,26,27]. Furthermore, the chemical composition of propolis varies depending on bee species, plant origin, and geographical locations, which can contribute to different treatment responses [20,28]. For example, a very low compositional overlap was reported (8~27%) between Brazilian and Chinese propolis, and a higher patch reaction was reported in the latter which may be attributed to the higher amount of caffeic acid and its derivatives [26,28]. Propolis in the temperate region is known to have more caffeic acid phenethyl ester (CAPE), one of the well-known components of propolis, while Brazilian green propolis has been claimed to contain no CAPE [20,21]. These data indicate much research remains to be performed before the therapeutic use of propolis in allergic patients.

We also found a higher prevalence of allergic rhinitis in vitamin D and probiotics users. Numerous studies have investigated the association between vitamin D, a fat-soluble vitamin that plays a crucial role in regulating the immune system, and allergic rhinitis. Clinical studies have demonstrated that vitamin D deficiency shifts the Th1/Th2 balance to Th2 in allergic rhinitis patients [29]. Also, in an interventional study conducted in 140 patients with moderate-severe allergic rhinitis in China, the efficacy of 4 weeks of vitamin D combination therapy with mometasone nasal spray was tested (control: mometasone only). As a result, better treatment response and quality of life were achieved in the treatment group [30]. However, a recent Mendelian randomization study failed to demonstrate the positive effect of serum vitamin D levels against the risk of allergic rhinitis. Therefore, more research is needed to confirm the association between vitamin D and allergic rhinitis [29]. Regarding the association between probiotics and allergic rhinitis, a randomized controlled study involving 425 participants showed that supplementation with *Lactobacillus paracasei* for four weeks significantly reduced nasal symptoms [31]. Additionally, it has notably improved the quality of life in patients with allergic rhinitis, particularly in ocular symptoms, compared to the placebo group [31]. However, not all studies have reported beneficial outcomes. Another double-blinded randomized control test was conducted in 173 adults with seasonal allergic rhinitis [32]. Patients have received a combination of *L. gasseri*, *B. bifidum*, and *B. longum* daily for 8 weeks (control: placebo) [32]. Although minor improvements in quality of life were observed in the probiotics group, there were no statistically significant differences in total nasal symptom scores [32].

This study has some inherent limitations. First, due to the inherent limitations of a cross-sectional, observational study, a causal relationship cannot be established. For example, the positive association between propolis use and the topic triad may be due to individuals with atopic conditions self-selecting propolis for symptom relief rather than propolis contributing to disease onset. Additionally, missing information on potential confounders, such as concurrent use of other herbal products, air pollution, or genetic data, raises the possibility of residual confounding.

Second, adults included in this study were significantly younger, had a higher socioeconomic status, and were less frail than adults excluded from the analysis, which limits the generalizability of our results to the entire adult population that may include more older, frailer, and less-resource abundant individuals. The potential selection bias suggests that our findings may be applicable to adults who are fit enough to participate in the extensive surveys process. Third, the study period included a pandemic period. The use of dietary supplements significantly increased during the pandemic due to the supposed immune-boosting effects of certain products [33]. Additionally, coronavirus disease 2019 (COVID-19) substantially increases the risk of asthma and other allergic diseases, which could have further influenced patterns of dietary supplement use [34]. To consider the differential impact of the pandemic, we stratified the regression analysis by years before and during the pandemic. However, this did not yield valid results due to insufficient sample size, and further exploration was reserved for future studies. Fourth, a more accurate depiction of the atopic triad may have been achieved if only allergic (atopic) asthma had been included. Some studies have implicated differences in the etiology of allergic and non-allergic asthma which could have affected its association with dietary supplements [35,36]. Currently, there is no universally accepted method to distinguish between the two phenotypes. The different mechanisms by phenotypes in the context of dietary supplement use can be further explored in future studies. Fifth, the KNHANES protocol limited the number of dietary supplements reported by participants to a maximum of four [14]. This restriction may hinder the analysis of their correlation with the atopic triad, potentially leading to missing data for individuals who simultaneously used a broader range of supplements. Lastly, because most data, including a history of comorbidities, supplement use, and diet, were self-reported, our findings may be prone to recall bias. To minimize this, the national surveys were conducted by trained interviewers who receive periodic training as part of the survey’s quality control program, which also aims to reduce recall-related problems [37].

Despite these study limitations, we found a significant association between propolis use and all three atopic conditions at a national level in Korean adults. The findings remained robust after rigorous adjustment for participant characteristics, including socioeconomic status, frailty, and dietary intake. Our findings highlight the need for further research on the use of certain supplements in allergic patients for evidence-based practice. More studies of quality are needed to understand the associations between dietary supplement use and the atopic triad.

## 5. Conclusions

In this study, we have found that more than 20% of the adults had a history of the atopic triad, and these adults were 1.6–2.0 times more likely to use propolis. Also, probiotics or vitamin D users were 1.21 times and 1.4 times more likely to have a history of allergic rhinitis, respectively. We did not find significant associations with the other supplements. Our findings highlight the need for further research on the use of certain supplements in allergic patients using quality data. Monitoring the safety and efficacy of dietary supplements in patients who preferred to use the dietary supplements may be considered. Future studies can further explore our study findings using longitudinal data.

## Figures and Tables

**Table 1:** Comparison of general characteristics of participants with or without any atopic triad in adults 19+ years old in KNHANES 2018–2021.

Variables	All		No Atopic Triad		Any Atopic Triad ^1^		*p*
Age							
19–29	2272	18.24 (0.47)	1516	15.42 (0.46)	756	28.90 (1.03)	<0.001
30–39	2575	17.35 (0.51)	1866	16.01 (0.52)	709	22.45 (0.97)	
40–49	3285	19.83 (0.48)	2608	20.14 (0.53)	677	18.64 (0.83)	
50–59	3424	19.95 (0.41)	2866	21.22 (0.46)	558	15.16 (0.71)	
60–69	3413	13.11 (0.33)	2978	14.47 (0.38)	435	7.97 (0.45)	
70+	3213	11.52 (0.37)	2822	12.74 (0.41)	391	6.89 (0.46)	
Year							
2018	5032	25.21 (0.65)	4096	25.62 (0.66)	936	23.63 (1.03)	0.044
2019	4955	24.99 (1.69)	4007	25.22 (1.70)	948	24.11 (1.91)	
2020	4006	24.49 (1.78)	3181	24.01 (1.76)	825	26.32 (2.08)	
2021	4189	25.31 (1.75)	3372	25.14 (1.74)	817	25.94 (2.02)	
Men	7822	49.96 (0.38)	6510	51.34 (0.43)	1312	44.74 (0.94)	<0.001
Living alone ^2^	5845	35.57 (0.61)	4463	33.34 (0.63)	1382	44.04 (1.13)	<0.001
High school or above	13221	80.95 (0.53)	10334	79.08 (0.59)	2887	88.04 (0.61)	<0.001
Living in MSA ^3^	7994	47.54 (1.02)	6440	47.32 (1.02)	1554	48.36 (1.46)	0.373
Low household income ^4^	3223	13.44 (0.47)	2725	14.01 (0.50)	498	11.30 (0.72)	<0.001
Medical aid ^5^	688	3.11 (0.21)	540	3.01 (0.21)	148	3.51 (0.40)	0.192
Current/past smoker	7056	42.69 (0.45)	5770	43.31 (0.49)	1286	40.35 (0.99)	0.007
Frequent drinking ^6^	2398	14.94 (0.33)	2006	15.71 (0.38)	392	12.04 (0.65)	<0.001
Frail ^7^	1344	5.10 (0.20)	1123	5.30 (0.22)	221	4.34 (0.39)	0.032
Supplement use, within a year	11396	61.83 (0.53)	9111	61.38 (0.57)	2285	63.53 (1.01)	0.045
Supplement use, current	10244	61.25 (0.77)	8201	60.95 (0.79)	2043	62.39 (1.19)	0.191
Number of supplements *	1.21 (0.02)		1.19 (0.02)		1.28 (0.03)		0.004
Multivitamins/minerals ^8^	4359	24.29 (0.41)	3476	24.12 (0.44)	883	24.94 (0.86)	0.363
Vitamin C ^9^	2059	11.62 (0.33)	1632	11.58 (0.36)	427	11.75 (0.62)	0.800
Omega-3 ^10^	2880	15.28 (0.37)	2324	15.36 (0.41)	556	14.99 (0.73)	0.667
Probiotics ^11^	2649	15.07 (0.40)	2015	14.26 (0.42)	634	18.14 (0.76)	<0.001
Red ginseng ^12^	980	5.14 (0.20)	791	5.15 (0.22)	189	5.11 (0.45)	0.937
Calcium ^13^	1167	5.95 (0.23)	947	6.05 (0.26)	220	5.60 (0.45)	0.368
Vitamin A/lutein ^14^	1245	6.65 (0.24)	1000	6.64 (0.27)	245	6.69 (0.49)	0.920
Propolis ^15^	388	2.10 (0.14)	287	1.89 (0.14)	101	2.90 (0.33)	0.001
Vitamin D ^16^	779	4.67 (0.21)	575	4.23 (0.23)	204	6.36 (0.49)	<0.001
Iron ^17^	167	0.96 (0.09)	134	0.92 (0.10)	33	1.13 (0.23)	0.359
Other vitamins/minerals ^18^	655	3.89 (0.19)	502	3.73 (0.21)	153	4.52 (0.43)	0.087
Any other	3389	18.06 (0.40)	2708	17.96 (0.44)	681	18.47 (0.76)	0.537

Unweighted frequency and weighted % (SE) are presented. *p*-values from the chi-square test between participants with or without the atopic triad. * Mean (SE) and *p* from Student’s *t*-test. ^1^ Ever diagnosed with asthma, allergic rhinitis, or atopic dermatitis. ^2^ Never married, separated, widowed, or divorced. ^3^ Metropolitan statistical areas (MSA), i.e., the top eight major cities in South Korea. ^4^ Equalized household income in the lowest 25% stratified by sex and age group. ^5^ Medical aid class 1 or 2, no health insurance, or unknown. ^6^ At least 4 times/month last year. ^7^ Frailty index ≥ 0.25 (range: 0~1, higher values indicate higher frailty). ^8^ Multivitamins with or without minerals. ^9^ Products to supplement vitamin C. ^10^ Products to supplement omega-3 fatty acids. ^11^ Products to supplement vitamin A or lutein. ^12^ Products to supplement probiotics. ^13^ Products containing red ginseng, its concentrated form or extract, excluding juice and decoctions. ^14^ Products to supplement propolis. ^15^ Products containing calcium and others that help calcium absorption or become bone components. ^16^ Products containing vitamin D only. ^17^ Products to supplement iron and hematopoietic components that may contain folic acid. ^18^ Products to supplement vitamins/minerals other than those above.

**Table 2:** Association between type of dietary supplements and the atopic triad from multivariable logistic regression analysis in adults 19+ years old in KNHANES 2018–2021.

Variables	Asthma	Allergic Rhinitis	Atopic Dermatitis	Any Atopic Triad ^1^
Multivitamins/minerals ^2^	1.03 (0.81–1.30)	1.02 (0.92–1.15)	0.87 (0.69–1.10)	1.01 (0.91–1.12)
Vitamin C ^3^	0.85 (0.60–1.21)	0.98 (0.84–1.14)	0.94 (0.69–1.27)	0.96 (0.83–1.10)
Omega-3 ^4^	0.90 (0.68–1.20)	1.06 (0.91–1.23)	0.93 (0.69–1.25)	1.03 (0.90–1.18)
Probiotics ^5^	0.97 (0.72–1.31)	1.21 (1.06–1.38)	0.94 (0.72–1.23)	1.17 (1.04–1.32)
Red ginseng ^6^	1.27 (0.84–1.93)	1.24 (1.00–1.54)	0.97 (0.58–1.63)	1.19 (0.98–1.45)
Calcium ^7^	0.98 (0.66–1.46)	0.93 (0.76–1.13)	1.09 (0.71–1.66)	0.97 (0.80–1.17)
Vitamin A/lutein ^8^	0.86 (0.56–1.31)	1.02 (0.84–1.23)	1.02 (0.69–1.53)	1.04 (0.87–1.24)
Propolis ^9^	1.90 (1.04–3.47)	1.64 (1.25–2.17)	2.04 (1.18–3.52)	1.57 (1.20–2.04)
Vitamin D ^10^	1.26 (0.79–2.01)	1.43 (1.16–1.75)	0.85 (0.53–1.36)	1.37 (1.12–1.67)
Iron ^11^	*-*	0.74 (0.44–1.23)	1.72 (0.78–3.81)	0.84 (0.53–1.34)
Other vitamins/minerals ^12^	0.67 (0.39–1.16)	1.03 (0.80–1.32)	1.27 (0.81–1.99)	1.02 (0.80–1.29)
Any other	1.10 (0.85–1.42)	1.11 (0.98–1.26)	1.16 (0.86–1.55)	1.12 (0.99–1.26)

Odds ratios (95% confidence intervals) are presented. Sampling weights were applied to account for the complex sampling design. The model was adjusted for age, sex, education, marital status, living area, household income, type of national insurance, smoking history, drinking frequency, and frailty. ^1^ Ever diagnosed with asthma, allergic rhinitis, or atopic dermatitis. ^2^ Multivitamins with or without minerals. ^3^ Products to supplement vitamin C. ^4^ Products to supplement omega-3 fatty acids. ^5^ Products to supplement vitamin A or lutein. ^6^ Products to supplement probiotics. ^7^ Products containing red ginseng, its concentrated form or extract, excluding juice and decoctions. ^8^ Products to supplement propolis. ^9^ Products containing calcium and others that help calcium absorption or become bone components. ^10^ Products containing vitamin D only. ^11^ Products to supplement iron and hematopoietic components that may contain folic acid. ^12^ Products to supplement vitamins/minerals other than those above.

## Data Availability

Data used in this study are publicly available for research purposes in a de-identified manner on the official KNHANES website at http://knhanes.cdc.go.kr (accessed on 10 April 2025).

## Supplementary Materials

The following supporting information can be downloaded at: https://www.mdpi.com/article/10.3390/medicina61040718/s1, Table S1. The 37-item frailty index. Table S2. Comparison of general characteristics by study inclusion in adults 19+ years old in KNHANES 2018–2021. Table S3. Comparison of the prevalence of the atopic triad and supplement use before and during the pandemic. Table S4. Comparison of general characteristics by a history of asthma. Table S5. Comparison of general characteristics according to allergic rhinitis. Table S6. Comparison of general characteristics by a history of atopic dermatitis. Table S7. Association between type of dietary supplements and atopic triad from multivariable logistic regression analysis with additional adjustment for diet in adults 19+ years old in KNHANES 2018–2021.

## Abbreviations

The following abbreviations are used in this manuscript:

CI	Confidence interval
COVID-19	Coronavirus disease 2019
IRB	Institutional Review Board
KNHANES	Korea National Health and Nutritional Examination Survey
MSA	Metropolitan statistical area
OR	Odds ratio
SE	Standard errors
