# Supplementary material for: Utilization of Dietary Supplements in People with the Atopic Triad in Korea: A Cross-Sectional Study Using KNHANES (2018–2021)
Source: Medicina (Kaunas). 2025 Apr 13;61(4):718. doi: 10.3390/medicina61040718 (PMC12028613; doi:10.3390/medicina61040718)
Supplement: Supplementary file 1 [file medicina-61-00718-s001.zip › medicina-3516559-supplementary.pdf]

Supplementary Tables:

Dietary Supplement Use in Adults with Atopic Triad in Korea: Insights from KNHANES (2018-2021)

Table S1. The 37-item frailty index

No	Item	Scoring
1	Ever diagnosed with a stroke	0= No; 1=Yes
2	Ever diagnosed with myocardial infarction	
3	Ever diagnosed with angina pectoris	
4	Ever diagnosed with rheumatic arthritis	
5	Ever diagnosed with osteoarthritis	
6	Ever diagnosed with osteoporosis	
7	Ever diagnosed with thyroid illness	
8	Diagnosed with depression within 1 year	
9	Diagnosed with any cancer within 5 years	
10	Any current restrictions in daily living/social activities due to illness, physical or mental disorder	
11	Stayed in bed for an entire day last month	
12	Self-reported health	0=Very good-good; 0.5=Normal; 1=Bad-very bad
13	History of hospitalization within 1 year	
14	Low physical activity (Metabolic equivalents (MET)-minutes/week, low 20%; adjusted by age, sex)	0= Not low; 1=Low
15	Unintentional weight loss (>3 kg) within 1 year	0= No; 1=Yes
16	Low body mass index	0=Not low; 1=Low (BMI <18.5 kg/m^2)
17	Obese	0=BMI <25 kg/m^2; 1=BMI ≥25 kg/m^2
18	Hypertension	0=No; 1=Yes (SBP≥140 mmHg or DBP≥90 or taking anti-hypertensive)<br>**If measurements were not available, survey data were used, i.e., ever diagnosed*
19	Hypotension	0=No; 1=Yes (SBP<90 mmHg or DBP<60)
20	Irregular heart rate	0=No; 1=Yes
21	Hypercholesteremia	0=No; 1=Yes (≥240 mg/dL after fasting 8 or more hours or taking lipid control medications)
22	Hypertriglyceridemia	0=No; 1=Yes (≥200 mg/dL after fasting 12 or more hours)
23	Low high-density lipoprotein	0=No; 1=Yes (male: <40 mg/dL, female: <50 mg/dL)
24	Diabetes	0=No; 1=Yes (Glucose ≥126 mg/dL after fasting 8 or more hours, or diagnosed with diabetes by a doctor, or taking glucose-lowering medication or administering insulin)<br>**If measurements were not available, survey data were used, i.e., ever diagnosed*
25	Hemoglobin A1c	0=Not high (≤5.6%); 0.5=Borderline (>5.6% and <6.5%); 1=Uncontrolled (≥6.5%)
26	Alanine transaminase	0: ≤ULN; 0.5: >ULN, ≤2x ULN; 1: > 2x ULN (ULN, male: >33 IU/L, female: >25 IU/L)
27	Aspartate transaminase	0: <ULN; 0.5: ≥ULN, <2x ULN; 1: ≥2x ULN (ULN, 20 IU/L)
28	Anemia	0=No; 1=Yes (Male: Hemoglobin <13 g/dL, Female: <12 g/dL)
29	Hematocrit	0=Not low; 1=Low (≤24%)
30	Renal disease	0=Not low; 1=Low (Cockcroft-Gault CrCl <60 mL/min)

		**If measurements were not available, survey data were used, i.e., currently have*
31	White blood cell	0=Not normal (4~10 thous/uL, inclusive); else 1
32	Red blood cell	0=Not normal (male: 4.2~6.3 million cells/uL, female: 4.0~5.4, inclusive); else 1
33	Platelet	0=Not normal (150~450 thous/uL, inclusive); else 1
34	Uric acid	0=Not high (<7 mg/dL); else 1
35	(Survey) Problems with chewing	0=Not at all/Not really/Okay; 0.5= Uncomfortable; 1=Very uncomfortable
36	(Survey) Problems with talking	
37	(Survey) Problems with hearing	0=No; 1=Yes

Table S2. Comparison of general characteristics by study inclusion in adults 19+ years old in KNHANES 2018–2021

Variables	All			Excluded[a]			Included			*p*
	n	%	SE	n	%	SE	n	%	SE	
Age										
19-29	2360	17.33	0.45	88	7.68	0.89	2272	18.24	0.47	<0.001
30-39	2719	16.78	0.48	144	10.70	1.00	2575	17.35	0.51	
40-49	3494	19.25	0.46	209	13.07	0.92	3285	19.83	0.48	
50-59	3687	19.74	0.39	263	17.54	1.16	3424	19.95	0.41	
60-69	3777	13.30	0.32	364	15.35	0.95	3413	13.11	0.33	
70+	4188	13.60	0.41	975	35.66	1.61	3213	11.52	0.37	
Year										
2018	5457	24.68	0.62	425	19.07	1.36	5032	25.21	0.65	<0.001
2019	5465	24.87	1.67	510	23.60	2.12	4955	24.99	1.69	
2020	4594	25.13	1.79	588	31.96	2.72	4006	24.49	1.78	
2021	4709	25.32	1.73	520	25.38	2.28	4189	25.31	1.75	
Men	11591	50.25	0.37	1231	52.52	1.38	10360	50.04	0.38	0.084
Living alone[b]	6643	35.75	0.59	798	37.65	1.37	5845	35.57	0.61	0.138
High school or above	13480	79.66	0.56	259	43.10	2.36	13221	80.95	0.53	<0.001
Living in MSA[c]	8795	47.36	1.00	801	45.44	1.99	7994	47.54	1.02	0.276
Low household income[d]	4067	15.13	0.50	844	33.94	1.51	3223	13.44	0.47	<0.001
National insurance, Medical aid[e]	920	3.64	0.23	232	9.27	0.88	688	3.11	0.21	<0.001
Current/past smoker	7756	42.65	0.42	700	42.22	1.29	7056	42.69	0.45	0.734
Frequent drinking[f]	2602	14.89	0.32	204	14.30	1.08	2398	14.94	0.33	0.573
Frail[g]	1563	5.60	0.21	219	18.97	1.45	1344	5.10	0.20	<0.001
Asthma	637	3.02	0.15	42	1.57	0.31	595	3.15	0.16	<0.001
Age at diagnosis, mean (SE)		35.48 (1.22)			55.93 (4.03)			34.53 (1.22)		<.0001
Allergic asthma[h]	118	0.80	0.09	6	0.26	0.12	112	0.86	0.09	0.010
Allergic rhinitis	2848	15.43	0.33	72	3.30	0.45	2776	16.57	0.35	<0.001
Age at diagnosis, mean (SE)		28.32 (0.38)			39.16 (2.66)			28.13 (0.38)		<0.001
Atopic dermatitis	639	3.96	0.18	24	1.34	0.34	615	4.21	0.19	<0.001
Age at diagnosis, mean (SE)		14.60 (0.64)			23.44 (4.05)			14.33 (0.64)		0.026
Atopic triad	3648	19.58	0.37	122	5.46	0.61	3526	20.90	0.39	<0.001
Any supplement use within a year	12446	61.09	0.52	1050	53.24	1.44	11396	61.83	0.53	<0.001
Any current supplement use	11161	60.51	0.76	917	52.42	1.66	10244	61.25	0.77	<0.001
Multivitamins/minerals[i]	4746	23.92	0.39	387	19.98	1.12	4359	24.29	0.41	<0.001
Vitamin C[j]	2207	11.33	0.32	148	8.20	0.75	2059	11.62	0.33	<0.001
Omega-3[k]	3155	15.13	0.36	275	13.51	0.90	2880	15.28	0.37	0.065
Probiotics[l]	2819	14.56	0.38	170	9.16	0.83	2649	15.07	0.40	<0.001
Red ginseng[m]	1049	4.99	0.19	69	3.37	0.47	980	5.14	0.20	0.003
Calcium[n]	1254	5.82	0.22	87	4.35	0.54	1167	5.95	0.23	0.015
Vitamin A/lutein[o]	1330	6.40	0.22	85	3.81	0.51	1245	6.65	0.24	<0.001
Propolis[p]	405	2.00	0.13	17	0.96	0.26	388	2.10	0.14	0.004
Vitamin D[q]	843	4.59	0.21	64	3.72	0.57	779	4.67	0.21	0.131

Iron[r]	180	0.95	0.08	13	0.77	0.24	167	0.96	0.09	0.487
Other vitamins/minerals[s]	705	3.82	0.18	50	3.01	0.50	655	3.89	0.19	0.121
Any other	3648	17.67	0.38	259	13.48	1.00	3389	18.06	0.40	<0.001
Number of supplements, mean (SE)		1.18 (0.02)			0.90 (0.04)			1.21 (0.02)		<0.001*

Unweighted frequency and weighted % (SE) are presented. *P*-values from the chi-square test between participants included or excluded, * indicates *p* from the student t-test.

a Excluded if < 19 years of age, did not participate in the dietary supplement surveys, or had missing covariate information.
b Never married, separated, widowed, or divorced.
c Metropolitan statistical areas (MSA), i.e., the top eight major cities in South Korea
d Equalized household income in the lowest 25% stratified by sex and age group
e Medical aid class 1 or 2, no health insurance, or unknown
f At least four times/month last year
g Frailty index ≥0.25, scores range from 0 to 1—the higher scores indicate higher frailty
h Age at diagnosis younger than 12 years of age
i Multivitamins with or without minerals
j Products to supplement vitamin C
k Products to supplement omega-3 fatty acids
l Products to supplement vitamin A or lutein
m Products to supplement probiotics
n Products containing red ginseng, its concentrated form or extract, excluding juice and decoctions
o Products to supplement propolis
p Products containing calcium and others that help calcium absorption or become bone components
q Products containing vitamin D only
r Products to supplement iron and hematopoietic components that may contain folic acid
s Products to supplement vitamins/minerals other than those above

Table S3. Comparison of prevalence of atopic triad and supplement use before and during the pandemic

Variables	2018-2019 (Pre-pandemic)			2020-2021 (During pandemic)			*p*
	n	%	SE	n	%	SE	
Asthma	314	3.18	0.22	281	3.13	0.22	0.880
Allergic rhinitis	1500	15.86	0.48	1276	17.30	0.52	0.040
Atopic dermatitis	313	3.83	0.26	302	4.59	0.29	0.049
Atopic triad	1884	19.88	0.54	1642	21.94	0.55	0.008
Any current supplement use	5146	49.59	0.75	5098	75.72	1.34	<0.001
Multivitamins/minerals	2269	22.21	0.56	2090	26.39	0.62	<0.001
Vitamin C	1021	10.33	0.44	1038	12.92	0.51	<0.001
Omega-3	1345	12.62	0.43	1535	17.96	0.59	<0.001
Probiotics	930	9.33	0.43	1719	20.86	0.60	<0.001
Red ginseng	502	4.71	0.26	478	5.59	0.31	0.029
Calcium	567	5.11	0.29	600	6.81	0.37	<0.001
Vitamin A/lutein	533	4.84	0.28	712	8.48	0.36	<0.001
Propolis	196	1.82	0.17	192	2.38	0.21	0.034
Vitamin D	355	3.74	0.25	424	5.62	0.35	<0.001
Iron	99	1.02	0.12	68	0.90	0.13	0.508
Other V/M	318	3.28	0.21	337	4.51	0.31	0.001
Any other	1563	14.87	0.48	1826	21.29	0.63	<0.001

Unweighted frequency and weighted % (SE) are presented. *P*-values from the chi-square test between participants included or excluded.

Table S4. Comparison of general characteristics by history of asthma

Variables	No asthma			Asthma			*p*
	n	%	SE	n	%	SE	
Age							
19-29	2180	18.05	0.47	92	24.11	2.43	<0.001
30-39	2496	17.36	0.51	79	17.21	2.05	
40-49	3228	20.12	0.49	57	10.91	1.57	
50-59	3349	20.16	0.42	75	13.50	1.70	
60-69	3301	13.09	0.34	112	13.63	1.44	
70+	3033	11.22	0.36	180	20.64	1.82	
Year							
2018	4871	25.23	0.65	161	24.66	2.10	0.947
2019	4802	24.96	1.69	153	25.91	2.97	
2020	3871	24.52	1.79	135	23.58	2.70	
2021	4043	25.29	1.75	146	25.85	2.81	
Men	10003	49.98	0.39	357	51.83	2.33	0.430
Living alone	5605	35.28	0.61	240	44.60	2.56	<0.001
High school or above	12862	81.20	0.53	359	73.26	2.07	<0.001
Living in MSA	7746	47.52	1.02	248	48.05	2.74	0.836
Low household income	3065	13.23	0.47	158	19.94	1.99	<0.001
National insurance, Medical aid	637	2.98	0.20	51	7.22	1.30	<0.001
Current/past smoker	6808	42.57	0.45	248	46.29	2.35	0.117
Frequent drinking	2351	15.13	0.34	47	9.18	1.52	0.002
Frail	1256	4.93	0.20	88	10.19	1.34	<0.001
Any supplement use within a year	11036	61.95	0.53	360	58.17	2.59	0.137
Any current supplement use	9926	61.39	0.77	318	57.17	2.77	0.109
Multivitamins/minerals	4219	24.33	0.42	140	22.95	2.12	0.523
Vitamin C	2000	11.70	0.34	59	9.19	1.39	0.111
Omega-3	2794	15.35	0.37	86	13.02	1.62	0.183
Probiotics	2566	15.11	0.41	83	13.79	1.72	0.463
Red ginseng	944	5.13	0.20	36	5.63	1.09	0.634
Calcium	1131	5.98	0.24	36	5.19	0.98	0.465
Vitamin A/lutein	1211	6.70	0.24	34	5.12	0.98	0.160
Propolis	370	2.06	0.14	18	3.31	0.94	0.103
Vitamin D	751	4.66	0.22	28	5.08	1.09	0.698
Iron	167	1.00	0.09	0	.	.	.
Other vitamins/minerals	637	3.93	0.19	18	2.66	0.68	0.129
Any other	3275	18.08	0.41	114	17.49	1.73	0.742
Number of supplements, mean (SE)	1.21 (0.02)			1.10 (0.07)			0.089*

Unweighted frequency and weighted % (SE) are presented. *P*-values from the chi-square test between participants with or without the diagnosis, * indicates *p* from the student t-test.

Table S5. Comparison of general characteristics by history of allergic rhinitis

Variables	No allergic rhinitis			Allergic rhinitis			*p*
	n	%	SE	n	%	SE	
Age							
19-29	1729	16.63	0.47	543	26.32	1.07	<0.001
30-39	1975	16.13	0.52	600	23.52	1.08	
40-49	2674	19.59	0.51	611	21.06	0.96	
50-59	2930	20.59	0.44	494	16.73	0.84	
60-69	3088	14.18	0.36	325	7.73	0.50	
70+	3010	12.89	0.41	203	4.64	0.41	
Year							
2018	4292	25.53	0.65	740	23.57	1.11	0.143
2019	4195	25.10	1.70	760	24.45	1.97	
2020	3365	24.16	1.76	641	26.19	2.15	
2021	3554	25.21	1.74	635	25.79	2.06	
Men	6838	51.31	0.42	984	43.20	1.07	<0.001
Living alone	4836	34.50	0.64	1009	40.98	1.20	<0.001
High school or above	10833	79.11	0.58	2388	90.24	0.62	<0.001
Living in MSA	6756	47.40	1.02	1238	48.23	1.55	0.519
Low household income	2908	14.22	0.50	315	9.53	0.69	<0.001
National insurance, Medical aid	585	3.06	0.21	103	3.36	0.43	0.473
Current/past smoker	6076	43.30	0.49	980	39.62	1.09	0.002
Frequent drinking	2087	15.50	0.37	311	12.13	0.73	<0.001
Frail	1205	5.39	0.22	139	3.62	0.39	<0.001
Any supplement use within a year	9541	61.14	0.57	1855	65.32	1.10	<0.001
Any current supplement use	8581	60.71	0.79	1663	63.95	1.27	0.008
Multivitamins/minerals	3638	23.98	0.44	721	25.84	0.98	0.065
Vitamin C	1702	11.45	0.36	357	12.46	0.72	0.180
Omega-3	2425	15.18	0.40	455	15.80	0.84	0.494
Probiotics	2112	14.24	0.41	537	19.27	0.88	<0.001
Red ginseng	825	5.08	0.21	155	5.46	0.53	0.499
Calcium	986	6.01	0.25	181	5.66	0.49	0.495
Vitamin A/lutein	1045	6.60	0.26	200	6.91	0.55	0.593
Propolis	300	1.88	0.13	88	3.21	0.38	<0.001
Vitamin D	606	4.24	0.22	173	6.87	0.55	<0.001
Iron	140	0.94	0.10	27	1.08	0.24	0.594
Other vitamins/minerals	527	3.73	0.20	128	4.71	0.49	0.052
Any other	2836	17.86	0.43	553	19.10	0.87	0.172
Number of supplements, mean (SE)	1.18 (0.02)			1.34 (0.03)			<0.001*

Unweighted frequency and weighted % (SE) are presented. *P*-values from the chi-square test between participants with or without the diagnosis, * indicates *p* from the student t-test.

Table S6. Comparison of general characteristics by history of atopic dermatitis

Variables	No atopic dermatitis			Atopic dermatitis			*p*
	n	%	SE	n	%	SE	
Age							
19-29	1968	16.55	0.46	304	56.54	2.39	<0.001
30-39	2448	17.17	0.51	127	21.63	2.03	
40-49	3226	20.32	0.49	59	8.77	1.24	
50-59	3381	20.55	0.43	43	6.26	1.02	
60-69	3372	13.54	0.34	41	3.43	0.58	
70+	3172	11.88	0.38	41	3.37	0.62	
Year							
2018	4872	25.28	0.65	160	23.51	2.13	0.174
2019	4802	25.11	1.70	153	22.22	2.41	
2020	3848	24.33	1.78	158	28.27	2.84	
2021	4045	25.28	1.75	144	26.00	2.74	
Men	7558	50.03	0.39	264	48.56	2.16	0.510
Living alone	5461	34.15	0.62	384	67.97	2.33	<0.001
High school or above	12674	80.39	0.55	547	93.68	0.90	<0.001
Living in MSA	7712	47.51	1.02	282	48.21	2.51	0.766
Low household income	3141	13.54	0.47	82	11.25	1.47	0.144
National insurance, Medical aid	668	3.14	0.21	20	2.45	0.64	0.344
Current/past smoker	6840	42.96	0.45	216	36.58	2.26	0.006
Frequent drinking	2326	15.07	0.34	72	11.98	1.45	0.057
Frail	1320	5.20	0.20	24	2.81	0.71	0.012
Any supplement use within a year	11041	62.07	0.54	355	56.39	2.34	0.014
Any current supplement use	9938	61.55	0.77	306	54.46	2.43	0.002
Multivitamins/minerals	4235	24.48	0.42	124	20.02	1.79	0.022
Vitamin C	1995	11.69	0.33	64	10.01	1.33	0.235
Omega-3	2810	15.48	0.37	70	10.77	1.41	0.004
Probiotics	2556	15.08	0.41	93	14.75	1.56	0.834
Red ginseng	956	5.23	0.21	24	3.19	0.81	0.051
Calcium	1138	6.02	0.24	29	4.42	0.89	0.126
Vitamin A/lutein	1209	6.71	0.24	36	5.17	0.94	0.152
Propolis	370	2.06	0.13	18	3.08	0.78	0.113
Vitamin D	753	4.68	0.21	26	4.58	1.02	0.928
Iron	158	0.93	0.09	9	1.78	0.70	0.101
Other vitamins/minerals	628	3.84	0.19	27	5.15	1.06	0.163
Any other	3297	18.22	0.41	92	14.43	1.71	0.047
Number of supplements, mean (SE)	1.22 (0.02)			1.03 (0.06)			0.002*

Unweighted frequency and weighted % (SE) are presented. *P*-values from the chi-square test between participants with or without the diagnosis, * indicates *p* from the student t-test.

Table S7. Association between type of dietary supplements and atopic triad from multivariable logistic regression analysis with additional adjustment for diet in adults 19+ years old in KNHANES 2018–2021

Variables	Asthma			Allergic rhinitis			Atopic dermatitis			Any atopic triad		
	OR	95%CI		OR	95%CI		OR	95%CI		OR	95%CI	
Multivitamins/minerals [a]	1.03	0.81	1.30	1.01	0.90	1.13	0.88	0.69	1.11	1.00	0.91	1.11
Vitamin C [b]	0.85	0.59	1.21	0.97	0.83	1.13	0.94	0.69	1.27	0.95	0.82	1.09
Omega-3 [c]	0.92	0.70	1.22	1.06	0.91	1.24	0.94	0.70	1.26	1.04	0.90	1.19
Probiotics [d]	0.97	0.71	1.32	**1.21**	**1.06**	**1.38**	0.92	0.71	1.21	**1.17**	**1.04**	**1.32**
Red ginseng [f]	1.28	0.85	1.93	1.23	0.99	1.53	0.97	0.58	1.61	1.19	0.98	1.45
Calcium [h]	0.98	0.65	1.45	0.92	0.75	1.13	1.08	0.70	1.66	0.97	0.80	1.17
Vitamin A/lutein [e]	0.85	0.56	1.30	1.01	0.83	1.22	1.00	0.67	1.49	1.03	0.86	1.23
Propolis [g]	**1.85**	**1.02**	**3.36**	**1.63**	**1.24**	**2.15**	**2.01**	**1.16**	**3.47**	**1.55**	**1.19**	**2.02**
Vitamin D [i]	1.25	0.78	2.00	**1.42**	**1.15**	**1.74**	0.84	0.52	1.35	**1.36**	**1.11**	**1.67**
Iron [j]	-	-	-	0.73	0.44	1.22	1.68	0.74	3.80	0.83	0.52	1.32
Other V/M [k]	0.66	0.38	1.15	1.03	0.80	1.32	1.24	0.79	1.95	1.02	0.80	1.29
Any other [l]	1.11	0.86	1.43	1.09	0.96	1.25	1.16	0.87	1.56	1.11	0.99	1.25

Odds ratios (95% confidence intervals) are presented. Sampling weights were applied to account for the complex sampling design. The model was adjusted for age, sex, education, marital status, living area, household income, type of national insurance, smoking history, drinking frequency, frailty, and the amount of nutrients from food (saturated fatty acids, omega-3, omega-6, cholesterol, carbohydrates, total dietary fiber, sugar, Ca, Fe, Na, β-carotene, retinol, thiamine, riboflavin, niacin, folate, vitamin C)

a Multivitamins with or without minerals
b Products to supplement vitamin C
c Products to supplement omega-3 fatty acids
d Products to supplement vitamin A or lutein
e Products to supplement probiotics
f Products containing red ginseng, its concentrated form or extract, excluding juice and decoctions
g Products to supplement propolis
h Products containing calcium and others that help calcium absorption or become bone components
i Products containing vitamin D only
j Products to supplement iron and hematopoietic components that may contain folic acid
k Products to supplement vitamins/minerals other than those above
